# Outlines of the Principal Diseases of Females; Chiefly for the Use of Students

**Published:** 1838-07

**Authors:** 


### Art. VI.

*Outlines of the principal Diseases of Females; chiefly for the Use of Students.* By Fleetwood Churchill, m.d., &c.; Physician to the Western Lying-in Hospital, (Dublin;) Lecturer on Midwifery, &c. in the Richmond-Hospital School of Medicine, &c.—*Dublin,* 1838. 8vo. pp. 402.

A work like the present has long been a desideratum in our medical literature, both for practitioners and for the student. The information which we possess on the diseases of women, is so scattered among a variety of monographs and journals in different languages; and much of it, however valuable, is so crude that it is scarcely available, even to the former class of readers, without much actual experience as well as study, and to the latter class it must, in many instances, be almost necessarily useless. The author informs us, in his preface, that "it has been arranged in the present volume that the *text* should contain an ample outline of the history, pathology, symptoms, and treatment of the diseases, without any detail of controversies or conflicting opinions, which are given in full in the *notes* appended to each page; so that the junior student, by confining his attention to the text, may acquire elementary information, which may be subsequently extended by consulting the notes and references." This is an excellent plan to go upon; because it not only affords a succinct view of the most approved opinions respecting the nature of these diseases, but makes the reader also, to a certain extent, familiar with the literature; a point which we hold to be of the highest importance, and which we think has not been sufficiently attended to in the education of the English medical students of the present day.

Dr. Churchill divides his work into two parts: 1st, the diseases of the external organs of generation; and, 2dly, the diseases of the internal organs. We cannot exactly agree with him as to the correctness of placing the diseases of the vagina under the second division, because, according to the most approved arrangement of these subjects, they belong to the first class: it is, however, a matter of no great importance, and we are far from feeling disposed to cavil about it. The symptoms, diagnosis, and treatment are distinctly arranged under separate heads; by which means reference to any peculiar point is greatly facilitated, and the immense mass of matter from which the author has had to selec his observations, better digested and condensed.

In describing "*Itching of the Vulva*," (chapter v.) the author mentions that it occasionally takes place "during pregnancy, from increase of fluids" in "the genital system;" but he makes no mention of that form so admirably described by Dr. Dewees, where the disease depends upon an aphthous state of the mucous membrane of the canal: perhaps, he intends to bring it under the head of Diseases of Gestation, which, he informs us, will be considered in a separate volume.

In his 6th chapter, on "*Inflammation of the Mucous Membrane of the Vulva*," he discusses the subject of infantile leucorrhœa. Having observed that it is occasionally met with as an epidemic catarrh, and described the celebrated case mentioned by Dr. Percival, of Manchester, where it was suspected to have been produced by criminal intercourse, he justly remarks that "the presence of this discharge is no proof whatever of such an offence." Circumstances, however, sometimes occur to render the diagnosis of this point extremely perplexing. We recollect a case of this sort where two sisters, the one six, the other four years old, were affected with this discharge, and where the extreme youth of the culprit would have led to the same conclusion, had not the discovery of well-marked phymosis placed the matter beyond doubt. Precisely similar circumstances, we know, occurred in the practice of one of our friends. The affection is well described by Dr. Churchill.

"The commencement of the disease is marked by local uneasiness, itching, and scalding on making water; the mucous membrane of the vulva is found inflamed and puffy, but for some time there is no discharge. The uneasiness felt by the child induces an attempt to relieve it by rubbing the parts; which, of course, aggravates the suffering and increases the inflammation. At a more advanced stage, there is observed a colourless, thin mucous discharge; speedily becoming more copious, thicker, and of a white or yellow colour. It is very often of an acrid character, and gives rise to a ring of inflammation, and sometimes of excoriation of the skin at the margin of the vulva. If the labia be separated, the mucous membrane will be found more vascular and of a deeper colour than usual; but in a very few cases does the inflammation extend up the vagina. The distress is increased with the progress of the disease; the smarting and scalding are very severe, and the little patient cannot walk without pain." (p. 11.)

In describing the symptoms of *acute Vaginitis* of adults, (p. 22,) which are given correctly, we think the author might have added pain in the back and loins, and throbbing in the pelvic region: they are, it is true, not constant, but nevertheless usually observed where the attack is severe; nor does he point out the analogy in the changes which the discharge undergoes, as the disease advances, to those which are observed in inflammation of the urethra, schneiderian membrane, &c. The gradual return to a more purulent, and at last merely mucous discharge, is not mentioned.

Although Dr. Churchill has described chronic leucorrhœa as resulting from chronic inflammation of the vagina, no mention is made of the simple leucorrhœa, so well described by Sir C. M. Clarke, resulting from debility and want of due tone and contraction in this canal. He briefly alludes to it in a note, in order to express his dissent from the correctness of these views, and doubts "whether general weakness and relaxation is any proof of debility of the vaginal mucous membrane." We cannot imagine any views more thoroughly confirmed by daily experience than those of the distinguished author to which we have just alluded. In large cities, the deranged and atonic state of the system, more especially of the chylopoietic viscera, is decidedly one of the most frequent causes of the above-mentioned form of leucorrhœa; which, in its turn, by increasing the general debility, aggravates the atony of these organs, the deranged state of which had primarily induced the discharge. From inattention to these circumstances can we alone explain the following observations, with the correctness of which we cannot possibly agree. "I have less experience," says Dr. C., "in general remedies, in consequence of the almost invariable success attending local treatment, consisting chiefly of different astringent solutions, thrown up the vagina by means of a syringe, or clyster-pipe and bladder." (p. 27.) We are surprised that no allusion has been here made to Dr. Locock's excellent article on *Leucorrhœa*, in the Cyclopædia of Practical Medicine, in which very different opinions are stated. Dr. Locock's observations on the treatment of the simple forms of leucorrhœa are well worthy of attention; and his directions for restoring the general tone of health are most judicious: and he concludes by saying that "local remedies are rarely required in these milder cases." We fully agree with these opinions; and can state, from pretty extensive experience, that, in most cases of simple leucorrhœa, the discharge will diminish in proportion as the tone and strength of the system is improved. We must equally dissent from the remark with which the author concludes this chapter: "The *consequence* of a long persistence of leucorrhœa is a relaxation of the parieties of the vagina, favoring the production of prolapsus uteri, and which can only be remedied by a diligent use of astringent injections." (p. 29.) Relaxation of the parietes of the vagina is seldom a *consequence* of leucorrhœa, but, in by far the majority of cases, leucorrhœa arises from relaxation of the vagina, the result of impaired health.

The next chapter is on "*Inflammation of the Glandular Structure of the Mucous Membrane covering the Cervix Uteri.*" After having quoted the symptoms of this affection, as given by Sir Charles Clarke, Dr. C. observes, "If, as Sir C. Clarke supposes, this state of the cervix always accompanied the white discharge, the disease would never be mistaken; but many cases occur in which the white discharge, exactly as described in the quotation above, is present without any puffiness or tenderness of the neck of the uterus." (p. 30.) He ought to have quoted the remark which this distinguished author has made a little further on, and to which Sir C. Clarke has expressly directed the reader's attention,—viz. that "even the *transparent* mucus of the vagina, when secreted in sufficient quantity to run down over the labia, (which have some motion over each other in the act of walking,) becomes also opaque and white. This change is attributable to the entanglement of air with mucus." Nor has Dr. Churchill paid any attention to a well-known variety in the appearance of the white mucous discharge, which we hold to indicate equally an inflamed state of the cervix: we mean where it is quite transparent, having the consistence of thin albumen; a fact observed by Dr. Locock, and we should have supposed familiar to every practitioner of any experience in these diseases. Sir C. Clarke makes a similar remark, viz. that "in many instances the white mucous discharge is much thicker than cream, having the tenacity of glue; and perhaps this is the state in which it comes away from the cervix uteri." Our own observations fully confirm this conclusion. The author states that "there are seldom any constitutional symptoms present," and in this remark he is to a certain extent supported by Sir C. Clarke: from very numerous cases, however, which have come under our notice, we cannot agree with this view; having found that the disease is almost invariably accompanied with much gastric derangement and general debility. The local symptoms are almost entirely omitted by Dr. Churchill. Dr. Locock correctly states that "there is much pain of the back, extending round the hips and down the thighs, and, though relieved, not removed, by the recumbent posture;" and Sir C. Clarke says, that it "is increased by whatever tends to call the neighbouring parts into action, such as riding, or by whatever produces pressure on the part affected. In this way the passage of a hard and large portion of fæces causes much distress; for not only are the blood-vessels filled in the act of expulsion, but, during the evacuation, constant, and sometimes considerable, pressure is made upon the cervix." Hence we find that patients of this class cannot sit down suddenly upon a hard seat or ride in a rough vehicle without considerable suffering.

In his treatment of *inflammation of the cervix uteri*, Dr. Churchill recommends abstracting blood, "either by venesection, leeches, or cupping to the loins;" and adds, in a note, "in almost all affections of the uterine system, I have found cupping the loins by far the most efficacious mode of taking away blood." (p. 30.) We could scarcely have supposed that an author who professes to write a book on the diseases of females, expressly compiled from the most approved authorities of the day, should have shown himself ignorant of one of the greatest improvements which have been made in the treatment of these affections: we allude to the practice of applying leeches, by means of a proper tube, to the os and cervix uteri, as recommended by Dr. Locock, and described by him in the article on Leucorrhœa already alluded to. If there be one uterine affection which requires this mode of locally abstracting blood more than another, it is this: we have for some years past used it in a very large number of cases, with the greatest relief. We do not agree with the author in regarding castor-oil as the best purgative in these cases, except, as Sir Charles Clarke observes, where the habit is very languid. The small doses of sulphate of magnesia which he recommends are infinitely better, as tending to allay irritation and assist in relieving the congested state of the neighbouring vessels. Neither do we consider a full dose of laudanum, with plenty of mucilaginous fluids for drink, the best means of relieving the frequent desire to evacuate the bladder: in many cases it is produced by acidity of the urine, the result of the gastric derangement; and in others, where it is produced more directly by the state of the cervix, it will subside as soon as we remove this latter condition. We regret that the author has paid so little attention to the consideration of this disease, which we hold to be one of the greatest importance, not merely from its frequency, but from the mischievous effects to which it too commonly leads. The whole chapter occupies only two pages.

Dr. Churchill divides the subject of *Prolapsus Vaginæ* (chapter v.) into two species; that of the anterior and posterior wall. In describing "*Prolapse of the anterior Wall of the Vagina*," he informs us "that the mechanism by which this is produced is sufficiently intelligible:" we could have wished that he had made it so. "The vagina (or, according to Siebold, the inner membrane only,) becomes relaxed from some cause, such as repeated child-bearing, and, the urine having been allowed to accumulate, it distends and forces the bladder downwards, protruding before it the yielding vagina. Every time that this accumulation takes place, the bladder is distended to a greater degree; so that that which at first occasioned no inconvenience gradually increases, until complete prolapse, or protrusion through the external orifice, is the result." (p. 34-5.) This is nothing more than a description of prolapsus vesicæ. Was ever a fluctuating tumour, protruding from the external parts, and formed by the distended bladder, called by any other name than prolapsus or procidentia vesicæ? It is, of course, covered by the vagina, as is the tumour in prolapsus uteri—but what of that?

In his preliminary observations on the *Pathology of the Uterus*, Dr. C. gives a very excellent remark respecting the condition of the vessels and nerves of the uterus after delivery: "the vessels, which were so much elongated, become tortuous, their coats are found thicker, and their caliber greater than natural; the nerves, also, though not so large as during pregnancy, remain of a considerable size and tortuous. The substance of the uterus does not return to the same density as before gestation, unless the interval be very long." (p. 53.) The author observes, in a note, "It is a remark, I believe, of the late Dr. Parry, of

Bath, that the tortuosity of the vessels is not a provision for some function they have yet to fulfil, but the result of some previous condition or some function already performed."

The observations on the utility of the *Speculum* are very just; the real value of vaginal examination is duly appreciated, and the limits pointed out within which this instrument can be used.

"We have seen that by the touch, in connexion with the local symptoms, we can obtain information on all points, except that of colour; and the accuracy of the knowledge so acquired is scarcely, if at all, inferior to that obtained by sight. It is very true that a delicate sense of touch and much experience is necessary before this degree of perfection will be attained, but it is equally certain that perseverance in availing ourselves of every opportunity (both on the living and dead body) will ultimately be crowned with success. The only deficiency in our means of diagnosis has been supplied of late years by the introduction of the *speculum;* and to this we undoubtedly owe the extension of our knowledge of uterine and vaginal diseases. Some new ones have been observed, and others, already familiar, have been more accurately described. There are, however, very considerable difficulties in the way of its use becoming common: it requires greater exposure, and is more revolting to feminine delicacy, than the other mode of examination; in some cases, also, it is much more painful. The information obtained by it is also much more limited, being confined to the state of the vagina and cervix uteri—still it is very frequently a most valuable adjunct." (p. 57-8.)

In describing that species of *amenorrhœa* which is produced by an imperforate condition of the os uteri and vagina, the author has entirely omitted to notice the admirable chapter on this subject in Richter's *Anfangsgründe der Wundarzneykunst,* which we hold to be by far the best account of any that has been given: indeed, we can scarcely imagine a more perfect specimen of well-arranged description, in the simplest language, than is exhibited in this valuable essay; and the author would not only have there found ample directions for the diagnosis and treatment of these cases, which occur under a great variety of forms, but might have quoted with advantage the fund of literature which this great author has added. The case by M. Amussat, which is given in a note, is highly interesting; but we cannot accept it as a substitute for what we have just referred to.

In the perusal of this volume, we have more than once had occasion to remark that the manner in which the different observations are put together is not calculated to render them always intelligible to a student, or give him clear notions upon the different subjects which are discussed. Compilation in many cases is allowed to be too evident; quotations are thrown together without sufficient care in arranging and blending them so as to appear as a whole. These remarks are suggested by the following observation respecting *dysmenorrhœa:* "Dewees," says Dr. C., "has tried the tinct. cantharid. with success, but the medicine upon which he appears to rely most confidently is the tinct. guaiaci, in doses of f℥ss three times a day. The pain is sometimes increased the first period after its exhibition, he says, but ultimately it affords complete relief." (p. 91.) Now, with regard to the tincture of guaiacum as a remedy in dysmenorrhœa, rather more than the simple fact is required to render the observation of any value. In the first place, it is *not* the plain tincture of guaiacum, but an ammoniated tincture, prepared after a peculiar formula; and, if the author had consulted Dr. Locock's article on this disease, he would have found that it was chiefly indicated in those cases where a rheumatic diathesis was present; a fact upon which the real value of this remedy in great measure depends, and which we have had opportunities of confirming.

We cannot recommend the arrangement which Dr. C. has adopted in discussing *menorrhagia:* it is not practical, and does not point out the two main conditions of the system under which this disease occurs, viz. the active and passive state: indeed, we must confess that we can see no real or important distinction between the three species which he has pointed out; and, even by his own account, the treatment in the first two species will be quite similar. The third form appears to be a state of local congestion, which he thinks that he has never observed in women under forty, or after the cessation of the menses. The usual signs of uterine congestion are correctly enumerated, and the ergot of rye recommended with much confidence; but we must beg to doubt the correctness of the opinion that the discharge in this form is produced by "the rupture of some of the vascular twigs which ramify on the lining membrane of the uterus." (p. 105.) The anæmic symptoms and effects of passive menorrhagia are indeed noticed, but not in the manner or to the extent which we could have wished. The author has alluded, in some comparatively unimportant points, to Dr. Locock's excellent article on this subject in the Cyclopædia of Practical Medicine, but the really valuable matter has been unnoticed.

The next chapter, (vii.) "*On the constitutional effects of the Disorders of Menstruation,*" seems to be intended as a sort of appendix to fill up the numerous gaps and defective points in those which precede it. When an author describes the symptoms, causes, consequences, diagnosis, and treatment (see Menorrhagia,) of a disease, we are surely justified in expecting that he has furnished a complete discussion of the subject. We do not deny that the chapter in question is well handled, and much valuable literature collected; but how can a student be expected to follow such an arrangement? A painter might almost as well attempt to pourtray a landscape by putting the foliage in one picture, and the ground, &c. in another; by such an arrangement the connexion between the disease and its effects upon the constitution is rendered obscure, and it becomes almost impossible for a beginner to form any clear views and general principles, or to make any useful or practical deductions.

Passing over several chapters we come to the 12th, "*On Moles.*" A considerable quantity of literature is collected upon this subject, but much has been omitted which ought to have been given: we allude more especially to the admirable essay on Diseases of the Placenta by Dr. J. J. Simpson, in the Edinburgh Medical and Surgical Journal. In speaking of the different species of mole, the author observes, "there is a variety of the fleshy mole which is worthy of distinct notice: it is figured in Denman's plates, in Granville's Illustrations of Abortion; and there is a specimen in the museum of the college of surgeons in this city, and another in Dr. Montgomery's museum. The texture of the ovum is much more dense than natural, especially the placental portion, which has very much lost its spongy feel; the membranes are unaltered, and, when opened, the inner surface of the placental portion consists of tuberculated projections of different sizes, from a pea to a walnut. Into one of these tubercles the cord is inserted, and the fœtus, in consequence, has perished." (p. 155.) This is not an uncommon species of mole, according to our experience. Although the author enumerates every variety of vesicular mole, yet we do not find it described as attaining the greatest bulk of these morbid growths; neither does he state distinctly that the hydatic mole increases in bulk with greater rapidity than the healthy ovum. The case of uterine hydatids, which is so graphically told by Dr. Gooch, in his work on some of the more important Diseases of Females, ought also to have been quoted.

Dr. Churchill's diagnosis of fibrous tumours of the uterus is very meager and imperfect: why not have quoted largely from Sir Charles Clarke on this subject, whose observations are so truly valuable and practical? Why omit the name of Mr. Ingleby, of Birmingham, whose excellent work on Obstetric Medicine teems with valuable cases, and not less valuable observations?

In chapter x., on "*Polypus of the Uterus*," although the author appears to have consulted the admirable works of Levret, Herbineaux, and Richter on this subject, he merely mentions the names of the first two authors, with some others, in giving a table of the comparative frequency of polypus at different ages. Dr. Hamilton's opinion that the hemorrhage which attends polypus is from the internal surface of the uterus, and not from the tumour itself, is very properly stated. We consider that this view holds good in some cases only; but, where the polypus has attained a considerable size, we must agree with Dr. Gooch in attributing the hemorrhage to the rupture of those engorged vessels which are observed upon the external surface. As soon as a polypus passes the os uteri, it is well known that it swells rapidly; and this, in all probability, must be chiefly attributed to the obstruction in its circulation by the pressure of the os uteri which encircles its neck. The chief vascularity of a fibrous polypus is, we presume, in the mucous membrane which covers it; and, in all probability, this is the seat of those ulcerations which we occasionally observe in the lower portion of large polypi, precisely as is seen in aggravated cases of prolapsus uteri. That these tumours, therefore, "are seldom or never attacked with inflammation or ulceration," (p. 188,) we must venture to doubt. We regret to say that the diagnosis of polypus uteri is meager and faulty in the extreme. Thus, for instance, the author states that it is distinguished "from *scirrhous enlargement* by the absence of pain and ulceration, and by the existence of a pedicle." (p. 193.) We would ask, are pain and ulceration always absent in polypus uteri? is the portion by which it is attached to the uterus always sufficiently within reach of the finger, or deserving the name of pedicle, to admit of being taken as the grounds of our diagnosis? Again, he observes that it is distinguished "from *prolapsus uteri* by there being no aperture (os uteri) or canal at the lower part of the tumour, by the detection of the os uteri in the pelvic cavity, and by the insensibility (generally) of the polypus." (p. 193.) Is no aperture ever found at the lower portion of a polypus? We admit that a practised finger will distinguish it from the os uteri in prolapsus, or we may ascertain it by passing a catheter into the opening; but why is this not mentioned? Is the condition of the vagina unworthy of notice, as a means of diagnosis between this displacement and polypus? Is the capability in most instances of returning prolapsed uterus, and with great relief to the patient's sufferings,—and the impossibility of doing so in polypus, producing great pain on every attempt to raise it, a point of so little importance as to be undeserving of attention? In describing the various instruments for applying the ligature upon a polypus, Dr. Churchill has totally omitted the one which has been invented by Baron v. Graefe, of Berlin, and which is decidedly the most perfect thing of the sort yet imagined: it is an improvement upon the double canula, and must be looked upon as one of the neatest of the modern surgical instruments. The omission of this is the more remarkable, as it has been for some time in the hands of many of the practitioners and instrument-makers of the metropolis.

We gladly pass on to a part of the work which we have read with much pleasure and satisfaction. The author's description of the appearance of the uterus in a case of corroding ulcer is excellent; indeed, we would almost say that, as far as our own reading goes, it is the best which we possess upon the subject.

"A *post-mortem* examination reveals clearly the nature and extent of the disease. The uterus is found more or less destroyed by ulceration, which sometimes extends itself circularly, so as to destroy the cervix and part of the body completely, leaving the remainder suspended by the ligaments, and unconnected with the vagina except by the surrounding cellular tissue; in other cases, it attacks the anterior or posterior wall of the uterus only, with the neighbouring portion of the vagina and the bladder or rectum. If the bladder be perforated, the vagina will be found more or less coated with matter deposited from the urine; if the communication be with the rectum, fæcal matter will be found in the vagina. I have never seen a case in which the bladder and rectum were both perforated. It is important to remark that there is no deposition of new morbid matter, either in the uterus itself or in the neighbouring parts. The portion of the uterus which remains undestroyed is slightly swollen and vascular." (p. 213-14.)

We cannot entirely agree with the author in his diagnosis of this disease from cancer, and we are inclined to side with Sir C. Clarke as to the essential difference of the pain in these two diseases: in other respects Dr. Churchill's diagnosis is well worthy of attention.

"The true ground of diagnosis, and the marked distinction between these two formidable complaints, is discovered by a *vaginal* examination. In cancer uteri, there is an extensive deposition into the cellular membrane and glands between the vagina and rectum, and between the vagina and the bladder, as well as into the substance of the uterus itself, connecting them so as to form one large mass, and *rendering the whole immoveable;* the finger, on being introduced into the vagina, finds *very little space*, and no power of *moving the parts with which it comes in contact.* Whereas, in corroding ulcer, no deposition having taken place, *the uterus can be moved by gentle pressure*, and part of the pelvic contents having been destroyed by ulceration, *there is more space than usual in the cavity.*" (p. 215.)

In discussing the questionable measure of excising the cervix uteri where the disease has not attacked the body of it, Dr. C. observes, "Caustic injections may be employed, or the ulcer touched with solid caustic, by means of the speculum. As yet, I have had no opportunity of trying this mode of treatment in cases sufficiently recent to afford reasonable expectation of benefit. I have used vaginal injections of nitrate of silver in advanced cases, with temporary relief: it assuaged the pain, and deprived the discharge of its fetid odour. Ten, twenty, or thirty grains, may be injected twice a day, dissolved in two or three ounces of water." (p. 217.) In this disease we can with confidence recommend to the author the application of leeches directly to the part by Dr. Locock's tube, as a valuable adjunct to other treatment. The whole chapter on this subject is well arranged, and shows much judicious reading and considerable experience.

Dr. Churchill gives a very fair summary of the literature and different opinions respecting *cancer uteri*. He inclines to the views of Dr. Copland and others, who consider that the fibrous portion of the cancerous mass which forms the septa results chiefly from an altered state of nutrition in the seat of the disease; whereas the soft semigelatinous portion which fills the cells formed by these septa, proceeds from a morbid secretion. We regret that the author has not noticed the fact that the majority of cases of cancer uteri are preceded by symptoms of inflamed cervix. Our own experience has taught us to believe that there are few cases which do not commence thus. He owns that, "in the latter part of the first stage, pressure on the cervix is occasionally painful," (p. 231;) but we should be much more inclined to say that, at the *beginning* of the first stage, it is almost invariably so.

The connexion between inflammation of the cervix and the early stages of scirrhus has excited much less attention that it ought to have done; which is the more to be wondered at as the former disease rarely exists to any extent without being attended by lancinating pains; the knowledge of which fact is of great importance in the treatment. We cannot agree with the author in saying that, " up to the actual commencement of ulceration, the discharge does not vary from the usual vaginal secretion, it is merely augmented in quantity." (p. 234.) If we carefully investigate the early history of these cases, we shall find that, in most instances, the discharge has exhibited either the creamy or albuminous characters which are peculiar to inflammation of the cervix. We are aware that, in this disease, if the inflammatory action be running to any extent, the discharge will become more or less watery; and this, we think, is most likely to take place when the disease is passing into the state of scirrhous induration.

The author's description of the effects produced upon the constitution by the progress of cancer uteri is very good; we think that, if he had quoted Sir C. Clarke's observations on this subject, he would have added to the value of the chapter. The diagnosis of the disease, however, is, in our opinion, given very unsatisfactorily. In regard to the treatment of cancer, we agree with the author in stating that iodine, as well as the preparations of iron, deserve a more extensive trial than they hitherto have had. To the value of chalybeate medicines we can decidedly bear a favorable testimony. The vaginal injections containing lead, which have been recommended by Dr. Leake, are very valuable when there is any inflammatory action present, especially if combined with some demulcent narcotic; after which, a salt of iron may be substituted for the lead with advantage. We regret that no mention whatever is made of leeches in the treatment of the first stage (*scirrhus*); their great importance we have already pointed out. In the second stage (*ulceration*), it is true, the author proposes that " a very few leeches may be applied, if necessary:" this, however, does not seem intended for the cervix uteri, but for the sacrum, which would be but of little use. We may add that, even in far-gone cases of cancerous ulceration of the uterus, we have found the application of leeches to the part invariably attended with relief, the pain being diminished, and the discharge assuming a more healthy appearance. Dr. C. informs us that he has "seen but little benefit from injections," (p. 246;) and yet his own statements lead to a different conclusion.

"Some time ago," he says, "I ordered injections of nitrate of silver (gr. x. to ℥i. of water twice a day) in a case of cancer, in hopes that it might arrest the ulceration; in this it failed, but I found that it afforded great relief in two particulars: first, it destroyed the excessive irritability of the ulcer, and diminished the pain; and, secondly, it entirely took away the fetid smell of the discharge: this latter effect was pointed out by the patient herself. I have tried it several times since, and always with the same good effect; I therefore feel justified in recommending it to the profession in this disease. The sympathetic and even distant pains which I have noticed (p. 233) are often and most effectually relieved by injections thrown up to the uterus; and, in the case of sciatica which has been mentioned, the injection of nitrate of silver was scarcely given before some mitigation was perceived, and after two or three more it ceased altogether for some time." (p. 246.)

The author has given an excellent digest of what has been published respecting excision of the neck of the uterus, and adds, when speaking of M. Lisfranc's operations, "it has been shown, however, by M. Pauly, that his operations (Lisfranc's) were fewer in number than was asserted; and that, so far from being either safe or successful, several died within twenty-four hours after the operation, and a considerable proportion (more than two-thirds,) were ultimately lost." (p. 248-9.) We think that M. Lisfranc owes to the profession a refutation of M. Pauly's most grave charges, if he deems the vindication of his honour a matter of any consequence.

The description of "*Retroversion of the Uterus*" is very confused, and the mechanism of it very imperfectly detailed. The names of Burns and Dewees do not once occur throughout this chapter.

In the chapter on "*Prolapsus Uteri*," the author quotes authorities in a way which must inevitably mislead the student. Was Capuron the first to show that it was produced by frequent parturition? Did Clarke first point out that uterine hemorrhage, or Jourdan that menorrhagia, might be causes of it, &c.? Where is the great name of Richter? why are his admirable observations unnoticed? The alteration which takes place in the character of the symptoms when the disease passes from the *partial* to the *complete* form, is not pointed out. The peculiar direction which the stream of urine takes, and many other circumstances are omitted; and we may say that, taken altogether, his diagnosis is meager and not altogether correct. The treatment of partial prolapsus, or procidentia, is very full. In cases of complete prolapsus, where, from swelling of the organ, it cannot be returned by venesection, purgatives, &c., the author proceeds at once to recommend "incisions into the substance of the womb." Here again we must complain of his omitting the application of leeches to the tumour. It is a most valuable remedy and one which, from repeated experience, we hold to be certain of reducing the size and hardness of the uterus, and rendering it capable of being returned; the objections of Sir Charles Clarke to the introduction of a sponge (p. 298, *note*,) ought to have been properly modified and explained away by showing that, if the sponge be wound round loosely with thread or fine string, and then soaked in the astringent fluid, it cannot dilate the vagina in the way it would do if allowed to expand.

Dr. Churchill's chapter on "*Inversion*," contains much valuable matter, collected from a considerable number of authors. In speaking of gradual inversion of the uterus from the attachment of a polypus to its fundus, he mentions Jourdan's name; altogether omitting that of Levret, who noticed, and even depicted this case at least seventy years ago; neither has he noticed the admirable observations of Dr. Dewees, in his compendious System of Midwifery, which contains more valuable matter on this subject in a short space than almost any work with which we are acquainted. Nevertheless, in justice to our author, we are bound to say that he has made amends for these omissions by the great extent and value of his quotations; the notes which he has appended to this chapter are numerous and highly interesting. His observations on the prognosis where there has been some delay are excellent. "But should the disease be of some days standing, are we to look upon the reduction as hopeless? Certainly not. There are cases on record of the attempt having been successful after days and weeks have elapsed: seventeen in Mr. White's case, twenty-four in Mr. Wynter's, twenty-seven in Mr. Dickenson's, three days in Mr. Cawley's, seven in Mr. Radford's (case 6), eight in MM. Chopart's and Ané's, eight in Mr. Ingleby's, ten or twelve in M. Lauverjat's, thirteen in M. Hoin's, and twelve weeks in Dr. Belcombe's." (p. 331.) We may add two interesting cases related by Boyer, where the uterus had resisted every endeavour to reduce the inversion, and which, in one case, remained fourteen days unreduced, and in the other more than eight years, and where, in consequence of a sudden and violent fall on the nates, reduction followed spontaneously and permanently.

The chapters on "*Diseases of the Fallopian Tubes and Ovaries*" are very excellent, and do the author much credit; he has quoted largely from our notice of Löwenhardt's Essay given in our second Volume, which contains much original and practical information. In speaking of encysted ovarian dropsy, he observes, "occasionally large veins are seen meandering over the surface of the tumour; but this is not generally the case. Arteries may also be felt pulsating sometimes; and in one case I observed a distinct *bruit de soufflet* like the placental *souffle*." (p. 364.) The literature on ovarian dropsy especially is very full; and the recent interesting cases of Messrs. Jeafferson and King, where the flaccid ovarian sac, after tapping, was drawn through the aperture and removed, are duly quoted.

Dr. Churchill's work is evidently the fruit of considerable labour; but in many cases this labour has not been so judiciously directed as could be wished. Authors of long standing and high reputation have been frequently omitted for those of later date, who have, in all probability, borrowed from them. It is but justice to say, however, that, with the exception perhaps of his diagnosis, wherever the author has given his own opinions apart from any quotations, they have been good and practical. The arrangement of his materials has not always been made with care or judgment: if we might so express ourselves, there is too little interstitial matter to blend them together and form a complete whole; and hence the value of the work to a student is considerably diminished. It is still, however, on the whole, a valuable work; and will prove useful to those who have not leisure or opportunity to consult more original writers. We doubt not that, when it reaches a second edition, the author will introduce many improvements into it; and also correct certain inaccuracies or peculiarities of expression, such as "the *accord* of symptoms," "a *grave* diagnosis," "*shedding*," for hemorrhage, &c. The work is a poor specimen of Dublin paper and print, but the price is proportionally small. Numerous typographical errors occur, and even the date on the title-page is put awry.

---

